# Types of Social Capital and Mental Disorder in Deprived Urban Areas: A Multilevel Study of 40 Disadvantaged London Neighbourhoods

**DOI:** 10.1371/journal.pone.0080127

**Published:** 2013-12-02

**Authors:** Marcello Bertotti, Paul Watts, Gopalakrishnan Netuveli, Ge Yu, Elena Schmidt, Patrick Tobi, Shahana Lais, Adrian Renton

**Affiliations:** 1 Institute for Health and Human Development, School of Health, Sports and Bioscience, University of East London, London, United Kingdom; 2 Research Fellow, Leeds Institute of Health Sciences, School of Medicine, University of Leeds, Leeds, United Kingdom; Hunter College, City University of New York (CUNY), CUNY School of Public Health, United States of America

## Abstract

**Objectives:**

To examine the extent to which individual and ecological-level cognitive and structural social capital are associated with common mental disorder (CMD), the role played by physical characteristics of the neighbourhood in moderating this association, and the longitudinal change of the association between ecological level cognitive and structural social capital and CMD.

**Design:**

Cross-sectional and longitudinal study of 40 disadvantaged London neighbourhoods. We used a contextual measure of the physical characteristics of each neighbourhood to examine how the neighbourhood moderates the association between types of social capital and mental disorder. We analysed the association between ecological-level measures of social capital and CMD longitudinally.

**Participants:**

4,214 adults aged 16-97 (44.4% men) were randomly selected from 40 disadvantaged London neighbourhoods.

**Main Outcome Measures:**

General Health Questionnaire (GHQ-12).

**Results:**

Structural rather than cognitive social capital was significantly associated with CMD after controlling for socio-demographic variables. However, the two measures of structural social capital used, social networks and civic participation, were negatively and positively associated with CMD respectively. ‘Social networks’ was negatively associated with CMD at both the individual and ecological levels. This result was maintained when contextual aspects of the physical environment (neighbourhood incivilities) were introduced into the model, suggesting that ‘social networks’ was independent from characteristics of the physical environment. When ecological-level longitudinal analysis was conducted, ‘social networks’ was not statistically significant after controlling for individual-level social capital at follow up.

**Conclusions:**

If we conceptually distinguish between cognitive and structural components as the quality and quantity of social capital respectively, the conclusion of this study is that the quantity rather than quality of social capital is important in relation to CMD at both the individual and ecological levels in disadvantaged urban areas. Thus, policy should support interventions that create and sustain social networks. One of these is explored in this article.

**Trial Registration:**

Controlled-Trials.com ISRCTN68175121 http://www.controlled-trials.com/ISRCTN68175121

## Introduction

The impact of socioeconomic disadvantages on mental health has been a growing area of research and, within this, the notion of social capital has been at the centre of significant academic and policy interest. Despite this, there is still a considerable debate as to whether social capital is a feature of individuals [1], groups [2] or both [3,4], the role of contextual measures of the physical environment in moderating the association between social capital and mental health, and the need for longitudinal analyses [5]. 

Social capital has been defined as ‘investment in social relations with expected returns’ (p. 30) [6]. The expectation is that such investment in social relations is beneficial for mental health [7] as it might reduce vulnerability to mental distress. However, various studies have shown that the different types of social capital are associated with mental health in different ways. The literature discusses several different types of social capital [8]. In this paper, we have focussed on its cognitive and structural components [9]. The cognitive component could be regarded as the ‘quality’ of social capital as it focuses on the types of social interaction between individuals [10,38]. It has been found to be positively associated with a range of mental health outcome measures across several countries at either the individual or ecological levels or both [5,10–12]. Conversely, the structural component could be regarded as the ‘quantity’ of social capital. It examines the extent and intensity of associational links between individuals, their behaviour and their participation to activities of the wider group. The evidence about the association between structural social capital and CMD is not conclusive [13]. For example, a systematic review by De Silva et al. [5] found a significant inverse association between measures of structural social capital and CMD at the individual-level but not at the ecological-level. At the individual-level, one study found a positive association between person’s level of group participation and CMD [14]. More recent studies have found either no association [10,12] or a negative association with CMD [11], albeit in the latter study, the sample included only older adults in Sweden and Finland. Many of these studies used measures of social capital based on a single question whilst we argue that there is a need for a more robust approach drawing on scales and dimensions of social capital. 

An additional interesting dimension that is particularly important in the context of deprived urban areas is the extent to which the physical characteristics of the local environment might moderate the association between types of social capital and mental health. For instance, crime – a physical characteristic of the neighbourhood - might encourage people to stay indoor preventing them from engaging in social networks or social support (as components of social capital), and, at the same time, increasing perceived stress which has an effect on mental health [39]. Thus, the physical characteristics of the environment might influence the strength between social capital and mental health. Only one study included features of the physical environment in examining the association between some measures of social capital and mental health. It found that ‘perceived neighbourhood greenness’ was more strongly associated with mental than physical health [15]. Yet, this study used self-rated rather than objective measures of the local physical environment. Thus, there appears to be lack of research on the moderating effect of the physical environment, a point echoed by a recent systematic review on the association between social capital and mental well-being in older adults [16].

In this context, the aims of this article are to examine: (1) the individual and ecological level association between cognitive social capital and CMD; (2) the individual and ecological level association between structural social capital and CMD; (3) the moderating influence of the physical characteristics of the local environment on the association between types of social capital and CMD; (4) the ecological level longitudinal change between some measures of social capital and CMD.

## Methods

### Study design

This study was nested within a cluster randomised trial, which examined the effectiveness of a complex community based programme that aimed to improve diet, physical and mental health in deprived communities in London. Between April 2011 and February 2012, a household survey of adults (16 years and above) was carried out at randomly selected addresses in 40 disadvantaged neighbourhoods of London [17] selected on the basis of the Index of Multiple Deprivation. The 20 London boroughs containing the most deprived 11% of Lower Super Output Areas (LSOAs) were identified. Within each of these 20 boroughs, the four most deprived LSOAs were selected and local authority and health professionals asked to choose two non geographically contiguous areas within the four identified in each borough. These were randomly allocated, one to intervention, the other to control condition. The data used for this article are based on the follow up survey for the trial and includes all the 40 LSOAs (intervention and control). Households were selected at random from the Post Office Address File for each of the 40 LSOAs. Some 150 addresses were randomly selected for fieldworkers to visit. Each of the 150 addresses was visited on five separate days and different times of the day, before being classified as a non-responding address. Ethical approval was obtained by The University of East London Research Ethics Committee (ref: ETH/07/96) in line with the declaration of Helsinki. Written informed consent was obtained from each participant to the study. For participants aged 16 or 17, written informed consent was obtained by parent or guardian. More information on sampling method are available on other published articles [18]. The follow up survey achieved a sample of 4,214 adults aged 16-97, 44.4% of whom were men. 

### Independent variables

We adopted a range of measures for cognitive and structural social capital and assessed the association between these and mental health at the individual and ecological-level. In line with other studies [18,19], ecological-level measures of social capital were derived by aggregating the individual-level scales and calculating the proportion of residents in each LSOA that reported high (5^th^ quintile) social capital. Strong Cronbach’s alpha correlations were obtained between all items in each scale for measures of cognitive and structural social capital. 

#### Cognitive social capital

In line with other studies [11,20,21], cognitive social capital was measured by examining a range of participants’ perceptions in relation to quality of neighbourhood and the number of types of social support. The social support scale has a long tradition in the psychological literature [22] and asks each participant three questions ‘how many people they could ask for help with groceries if they are unwell’, ‘with money’ and ‘for advice in time of crisis’. As such, it is a measure of the quality of interactions between individuals. It can be best understood in opposition to ‘social networks’ that measures the number of ties individuals have outside the family. We adopted a binary score (0=none; 1=one or more) for the three questions and combined them into a single scale. Cronbach’s Alpha for this scale (0.90) shows excellent internal consistency.

‘Perception of neighbourhood quality’ includes various questions about the quality of the local physical infrastructure (attractiveness of buildings and environment, peacefulness, quality of parks, opens spaces and children’s play areas) and the extent to which the neighbourhood is affected by social unrest such as drunkenness, rubbish, vandalism, drugs, and teenagers hanging around in the street. All but one of these items were measured using a 5 points Likert scale which was dichotomised and combined into a single scale. Consistency for this scale was good (Cronbach’s Alpha = 0.85). Questions were drawn from the World Values survey [23] and also asked in the Standard Eurobarometer [24] survey carried out by the European Commission in every country since the early 1970s.

#### Structural social capital

Structural social capital represents individual social networks and participation in the local neighbourhood. Measures of structural social capital chosen for analysis in this study are: a) type and frequency of social networks and; b) civic participation. The ‘social networks’ scale was derived from questions in the social capital harmonised questionnaire [25] set and have been used in a variety of studies on social capital [5,21]. Respondents were asked the frequency with which they meet up, speak to phone and write to relatives and friends (excluding people the participant lives with) and the frequency with which they speak to neighbours, using a 5 point Likert-style scale (from ‘never’ to ‘most days’). Each Likert-style scale item was dichotomised and these items were combined into a single scale. The internal consistency of this scale was acceptable (Cronbach’s alpha = 0.75). 

To construct the ‘civic participation’ scale respondents were asked about their attendance (yes/no) to activities, events, actions in an attempt to solve a problem affecting local people and volunteering in the last 12 months. Items were combined into a single scale and items that did not correlate well with other scale items were dropped. The items that were dropped asked respondents about attending events at nightclubs or volunteering at ‘other’ events. The internal consistency of the final scale used for analyses was good (Cronbach’s alpha= 0.80).

#### Neighbourhood measures

In order to capture the characteristics of the physical environment, we used a measure of social disorder and incivilities created through systematic social observation of the 40 neighbourhoods by trained observers. Items used follow validated tools [26,27] and included: presence and amount of: litter and broken glass; graffiti; vandalised facilities; broken windows; security measures; unattended dogs; large items dumped in public areas; dog foul; needles/syringes/condoms; empty alcohol bottles/cans; signs of home personalisation; greenery; and neighbourhood watch signs. The reliability of measure between the trained observers was good. Each item on the scale showed less than 10% variability between raters. The number of each of these items in each neighbourhood were recorded and combined into a continuous scale, adjusted for the size of each neighbourhood. 

### Dependent variable: Common Mental Disorder (CMD)

We included the GHQ-12 (General Health Questionnaire) as measure of mental disorder. It assesses each respondent’s current state and asks if that differs from their usual state. The underlining principle of GHQ-12 is that it allows assessment of whether an individual is affected by a range of mental disorders without diagnosing them. The relationship between mental disorder measured by GHQ-12 and social environment has been shown in a number of studies [31]. GHQ-12 measures three distinct constructs of ‘anxiety’, ‘social dysfunction’ and ‘loss of confidence’ [28]. In this study, GHQ-12 is used as a continuous variable, the binary measure is >/= 3 on the 12 point scale. 

### Analysis

We conducted a multilevel analysis of 4,214 people nested within 40 deprived London neighbourhoods (Lower Super Output Areas). All analyses were conducted using complete cases, defined as those survey respondents with data for all socio-demographic variables used for adjustment and for the outcome of interest. All models were adjusted for age, gender, education and ease of managing on income as potential confounders of individual and ecological-level associations between cognitive/structural social capital and CMD (Table 1). Multilevel modelling in Stata v11 [29] was used to examine associations between each domain of individual and ecological-level social capital (independent variables) and CMD (outcome measure: GHQ-12) separately (table 2). Model 1 and model 2 measure the association between cognitive/structural social capital respectively and CMD at the individual-level. Model 3 examines both cognitive and structural social capital at the individual and ecological levels in the same model in response to a common criticism of previous studies [8] which reported that individual-level measures of social capital may affect ecological-level measures. 

**Table 1:** Descriptive statistics for socio-demographic and social capital measures.

**Individual-level factors**	n	%		
**Sex**				
Male	1,871	44.4		
Female	2,343	55.6		
**Age**				
16-24 years	996	23.64		
25-34 years	1,102	26.15		
35-44 years	880	20.88		
45-54 years	580	13.76		
55-64 years	362	8.59		
65+ years	294	6.98		
**Ease of managing on income**				
	Very easy	230	5.91	
	Fairly easy	1,008	25.92	
	Neither easy nor difficult	681	17.51	
	Fairly difficult	981	25.22	
	Very difficult	989	25.43	
**Highest level of education**				
Primary	440	10.54		
Secondary (GCSE or equivalent)	1,287	30.83		
Further ('A' Level or equivalent)	968	23.19		
Higher (University degree)	1,384	33.16		
Other	95	2.28		
**Social Capital Variable**	Mean	SD	Min	Max
**Individual-level Cognitive Social Capital**				
Social Support Scale (0-3)	2.13	1.24	0	3
Perception of neighbourhood quality Scale (0-10)	6.22	3.13	0	10
**Individual-level Structural Social Capital**				
Social Networks Scale (0-24)	18.69	4.85	0	24
Civic Participation Scale (0-29)	2.82	3.19	0	29
**Ecological-level cognitive Social Capital**				
% in top quintile for social support	8.64%	0.08	0.00%	29.13%
% in top quintile for Perception of neighbourhood quality	28.36%	0.16	3.33%	75.00%
**Ecological-level Structural Social Capital**				
% in top quintile for social networks	21.40%	0.16	0.00%	62.04%
% in top quintile for civic participation	15.28%	0.14	0.00%	58.10%
**Outcome Variable**				
GHQ-12 (General Health Questionnaire)	0.66	1.63	0	12

**Table 2:** Association between cognitive/structural social capital and CMD at the individual and ecological levels.

	Null Model	Model 1		Model 2		Model 3	
		Odds Ratio	CI	Odds Ratio	CI	Odds Ratio	CI
**INDIVIDUAL-LEVEL**							
**Cognitive Social Capital**							
Social support		0.97	0.85-1.10	1.09	0.96-1.25	1.09	0.95-1.24
Perceptions of neighbourhood quality		0.96	0.92-1.01	0.96	0.92-1.01	0.97	0.92-1.01
**Structural Social Capital**							
Social Networks		**0.87**	**0.85-0.89**	**0.87**	**0.84-0.89**	**0.87**	**0.84-0.89**
Civic Participation		**1.11**	**1.07-1.14**	**1.11**	**1.06-1.15**	**1.11**	**1.06-1.15**
**ECOLOGICAL-LEVEL**							
**Cognitive Social Capital**							
Social support						0.21	0.01-3.15
Perceptions of neighbourhood quality						0.47	0.12-1.81
**Structural Social Capital**							
Social Networks						**0.19**	**0.04-0.87**
Civic Participation						0.65	0.13-3.17
AIC	2331.16	1701.24*; 2097.84**		1552.75		1555.37	

All models adjusted for age, gender, education and ease of managing on income; Significant associations (p= <0.05) in **bold;** (*) AIC for Individual-level Cognitive social capital; (**) AIC for Individual-level Structural social capital

Model 1: Individual-level Cognitive and Structural social capital examined independently

Model 2: Individual-level Cognitive and Structural social capital examined in the same model

Model 3: Ecological-level and Individual-level Cognitive and Structural Social capital

Intra class correlation coefficient for the null model (adjusted adjusted for age, gender, education and ease of managing on income) = 0.07

Median odds ratio for the null model (adjusted adjusted for age, gender, education and ease of managing on income) = 1.6

Additional multilevel models with interaction terms were fitted to examine whether relationships between individual-level social capital and CMD were moderated by incivilities measured at the ecological-level (Table 3). Model 4 introduces a measure of the characteristics of the physical environment (neighbourhood incivilities) and examines the association between types of social capital and CMD after having adjusted for neighbourhood incivilities. Model 5 adds multiplicative interaction terms to model 4 for each measure of social capital and ecological-level incivilities (social capital x ecological level incivilities). The subgroup analysis (Table 4) explores the moderating role of neighbourhood incivilities and the nature of these interactions by examining the association between types of social capital and CMD in high and low incivilities neighbourhoods separately (model 6). 

**Table 3:** Stratified analyses of the extent to which neighbourhood incivilities moderate types of social capital.

	Null Model	Model 4		Model 5	
		Odds Ratio	CI	Odds Ratio	CI
**Individual-Level Cognitive Social Capital**					
Social support		1.09	0.96-1.25	1.06	0.92-1.22
Perceptions of Neighbourhood quality		0.96	0.92-1.01	0.99	0.94-1.05
**Individual-Level Structural Social Capital**					
Social Networks		**0.87**	**0.84-0.89**	**0.87**	**0.84-0.90**
Civic Participation		**1.11**	**1.06-1.15**	**1.09**	**1.05-1.14**
**Neighbourhood Incivilities**					
Low		1.00		1.00	
High		0.91	0.76-1.09	**0.41**	**0.21-0.79**
Social support x incivilities				1.24	0.83-1.87
Perception of neighbourhood x incivilities				**0.79**	**0.68-0.91**
Social Networks x incivilities				0.95	0.88-1.03
Civic Participation x incivilities				1.08	0.98-1.20
AIC	2331.16	1553.68		1545.39	

All models adjusted for age, gender, education and ease of managing on income; Significant associations (p= <0.05) in **bold**

Model 4: Individual-level Cognitive and Structural Social Capital controlling for Neighbourhood IncivilitiesModel 5: Individual-level Cognitive and Structural Social Capital controlling for Neighbourhood Incivilities and Interaction terms

**Table 4:** Subgroup analyses: association between social capital and CMD in low and high incivilities neighbourhoods.

		Model 6					
		Low Incivilities Neighbourhoods			High Incivilities Neighbourhoods		
**Subgroup Analyses**	Null Model	Odds Ratio	CI		Odds Ratio	CI	
**Individual-Level Cognitive Social Capital**							
Social support		1.06	0.92-1.22		1.23	0.83-1.84	
Perceptions of Neighbourhood quality		0.99	0.94-1.05		**0.77**	**0.67-0.89**	
**Individual-Level Structural Social Capital**							
Social Networks		**0.87**	**0.84-0.90**		**0.81**	**0.75-0.88**	
Civic Participation		**1.09**	**1.05-1.14**		**1.19**	**1.08-1.31**	
AIC	2331.16	1880.81			1555.37		

All models adjusted for age, gender, education and ease of managing on income; Significant associations (p= <0.05) in **bold**

Finally, we examined associations between longitudinal change in ecological-level social capital and individual-level CMD from the 2011-12 survey described above (table 5). This was possible due to data available from a previous baseline survey undertaken between February 2008 and August 2009 on a different sample of 4,107 individuals but in the same 40 neighbourhoods. The same questions were asked about social networks and social support as measures of cognitive and structural social capital respectively. The analysis was produced by calculating the difference between each ecological-level social capital measure in the main survey and comparable measure in the previous survey. Model 8 examines associations between longitudinal change in ecological-level social support and social networks and CMD measured by GHQ-12 at the follow up survey. Model 9 examines the same longitudinal change as in the previous model, after controlling for individual-level cognitive and structural social capital. An intra-class correlation coefficient was calculated to provide information about the proportion of variance in CMD that can be attributed to variance at the individual and ecological levels. We have also presented a median odds ratio [37] to quantify the variance at the ecological level. 

**Table 5:** Longitudinal change in the association between cognitive/structural social capital and CMD at the ecological-level.

**LONGITUDINAL CHANGE**	Null Model	Model 8		Model 9	
		Odds Ratio	CI interval	Odds Ratio	CI interval
**ECOLOGICAL-LEVEL**					
**Cognitive Social Capital**					
Social support		1.04	0.23-4.62	0.45	0.10-1.99
**Structural Social Capital**					
Social Networks		**0.12**	**0.03-0.53**	0.37	0.08-1.58
**INDIVIDUAL-LEVEL AT FOLLOW UP**					
**Cognitive Social Capital**					
Social support				1.10	0.98-1.23
**Structural Social Capital**					
Social Networks				**0.87**	**0.8-0.90**
AIC	2331.16	2327.52		1880.81	

All models adjusted for age, gender, education and ease of managing on income; Significant associations (p= <0.05) in **bold**

Model 8: Ecological-level Cognitive and Structural social capital Model 9: Ecological-level Cognitive and Structural social capital controlled for Individual-level cognitive and structural social capital at follow up

## Results

The multilevel regression analyses have enabled us to examine the influence of different types of social capital (cognitive and structural) at the individual and ecological levels. All associations have been controlled for age, gender, education, and ease of managing on income. As table 2 shows, four models were developed with variable but good model fit in relation to the null model as measured by AIC (Akaike Information Criterion).

Table 1 provides the main socio-demographic variables and social capital measures used in the sample. The sample is young, slightly more female, fairly well educated but finds it difficult to manage on the income available to them. In particular, it is characterised by a slightly higher proportion of female respondents and over 70% of the population are under 44 years of age. Some respondents find it ‘fairly’ or ‘very difficult’ to manage on income. Yet, the majority of them have at least an ‘A’ level or equivalent. 

The mean for the Social Support scale is quite high showing that on average respondents received two types of social support from at least one person. However, the standard deviation is rather high showing that the distribution of responses was considerably scattered. 

Perception of neighbourhood quality shows that on average respondents found a ‘very’ or ‘fairly good’ quality of the neighbourhood. The distribution around the mean is however scattered suggesting a significant variation in responses across the sample. The social networks scale shows a high mean which reflects that most respondents meet, speak or write to their relatives and friends at least once or twice a month. The ‘Civic Participation’ scale shows an extremely low mean (2.82) with a standard deviation (3.19) which means that the overall distribution is skewed. This means that very few respondents are actively engaged in activities, events or actions in an attempt to solve a problem affecting local people and volunteering in the last 12 months.

The intra-class correlation coefficient for the model adjusted only for age, gender, education and ease of managing on income was 0.07, suggesting that 7% of the variation in CMD is attributable to differences at the ecological-level. The median odds ratio for the same model was 1.6. This suggests that if an individual moved to a randomly selected neighbourhood where there was on average a higher probability of experiencing CMD, their median increase in odds of experiencing CMD would be 1.7). 

Table 2 shows associations between individual and ecological-level measures of cognitive and structural social capital and CMD as measured by GHQ-12. Model 1 examines individual-level cognitive and structural social capital independently from each other. There are significant associations between structural social capital and CMD at the individual-level, however the direction of association for the two components of structural social capital is conflicting. Higher ‘social networks’ are associated with lower CMD, whilst higher ‘civic participation’ is associated with higher CMD. The association with social networks suggests a reduction in the odds of CMD by 13% for each one point increase on the social networks scale. The association with civic participation is similar in magnitude but in the opposite direction. In Model 2 these associations are broadly the same after mutually adjusting for individual cognitive and structural social capital and in Model 3 also largely the same when ecological and individual-level cognitive and structural social capital are examined in the same model. In Model 3 there is a negative association between the ecological-level social networks component of structural social capital and CMD, but no association between the ecological-level civic participation component and CMD. The picture is rather different for individual and ecological-level cognitive social capital as none of the components are associated with CMD in models 1, 2 or 3.

Tables 3 and 4 examine the extent to which the physical characteristics of the neighbourhood studied- measured by neighbourhood incivilities -moderates the associations between types of social capital at the individual-level and CMD. The association between cognitive social capital and CMD in table 3 is non-significant consistently with table 2. The direction of association between structural social capital and CMD is also consistent with table 2, thus the components of structural social capital are significantly associated with CMD after adjustment with neighbourhood incivilities and when interaction terms are introduced in the model. As in table 2, their association with CMD is negative in relation to social networks and positive in relation to civic participation. It is surprising to note that there is a negative association between neighbourhood incivilities and CMD, when interaction terms are introduced in the model (model 5, table 3). Thus, it would appear that respondents living in neighbourhoods characterised by high incivilities have 59% lower odds of experiencing CMD than if they lived in neighbourhoods with low levels of incivilities. Finally, there is a significant negative association between the interaction of contextual measures of the physical environment (neighbourhood incivilities) and perception of neighbourhood quality and CMD. This suggests that both neighbourhood incivilities and perceptions of neighbourhood quality interact to influence mental health.

The nature of this interaction can be seen more clearly in table 4 which provides a subgroup analyses differentiating between high and low incivilities neighbourhoods. Perception of neighbourhood quality is negatively associated with CMD in areas of high incivilities, but not associated in low incivilities neighbourhoods. This means that in high incivilities neighbourhoods, there is a significant and negative association between perceptions of neighbourhood quality and CMD. Structural social capital maintains the same direction of association as in previous tables and does not seem to vary according to level of incivilities. 

Table 5 shows the extent to which the longitudinal changes in selected measures of social capital at the ecological-level are associated with CMD. Model 8 shows that longitudinal changes in ecological-level social networks are negatively associated with CMD. However, this association is no longer present when individual-level cognitive and structural social capital at follow up are included in the following model (model 9). This means that once individual-level structural social capital is introduced in the analysis of longitudinal change, the ecological level association between structural social capital and CMD disappears. 

## Discussion

The main finding from this study is that social networks, a component of structural social capital, is significantly and negatively associated with mental disorder. The direction of this association is maintained at both individual and ecological levels and also when the moderating effect of the physical environment is included into the model. In contrast, cognitive social capital is not significantly associated to CMD. 

Our analyses suggest that social networks are important for mental health. This means that the more written, face to face and telephone contacts individuals have with relatives and friends, the lower the odds of experiencing CMD. Moreover, a negative association between social networks and CMD at the ecological-level exists even when individual measures are included in the model and leads to conclude that there is an area effect of social capital as theoretical contributions from notable scholars suggest [3]. 

When contextual measures of the characteristics of the physical environment are introduced into the model (neighbourhood incivilities), the association between social networks and CMD is still significant and negative in both high and low incivilities neighbourhoods. Thus, it can be concluded that some physical characteristics of the neighbourhood (i.e. neighbourhood incivilities) do not moderate the association between social networks and CMD. This point is echoed by a study looking at the association between individual and neighbourhood characteristics and mental ill health [19]. 

Moreover, individual perceptions of neighbourhood quality are significantly and negatively associated with CMD in high incivilities areas but are not in low incivilities areas. This means that individual perceptions of neighbourhood are only important in explaining mental health in high incivility areas rather than in low incivility areas. Thus, only when individuals live in high incivility areas, their perception of neighbourhood quality is important for their mental health. It appears that the interaction between perception and contextual measures of the physical environment are important for mental health.

A common explanation of the negative association between social networks and CMD is reverse causality. This would support the argument that people with lower or no common mental disorders are more likely to engage in contact with relatives and friends. We introduced a longitudinal analysis in the study to examine this argument in more detail. Although our longitudinal examination shows a negative association between ecological-level social networks and CMD, when individual-level social networks are introduced in the model, the significant association disappears. Thus, our conclusion is that the reverse causality argument might well apply. 

In our study social networks and civic participation are both significantly associated with CMD but the sign of correlation is of opposite direction. This suggests that more research needs to be done on the utility of the structural component of social capital. Alternatively, social networks and civic participation and their associations with CMD could be discussed separately rather than as a single structural component. 

One of the reasons for the positive association between civic participation and CMD might be found in the argument that people who attend events concerning ‘actions to solve a problem affecting the local area’ have directly been victims of such issues and consequently more likely to have experienced some mental health problems to start with. An alternative explanation draws upon the idea that the ‘act’ of attending these events might lead to develop mental health problems. Although some other studies found no impact of civic participation on depression [12,14], our evidence is in line with similar findings from a multi-level analysis of social capital and mental health in Japan [13]. 

An additional important finding is the non-significant level of association between cognitive social capital and CMD at the individual or ecological-level. This is at odds with most other studies which have consistently found a negative association between cognitive social capital and CMD [5,30] and reinforced further by the evidence from a London based study examining a mix of low and high SES neighbourhoods which used social support as independent measure and GHQ-12 as outcome variable [31]. This difference might be explained by the different measures of cognitive social capital used in this study. For example, other studies [13] use perceived trust to measure cognitive social capital. In this study, we had an item that asked respondents about the proportion of neighbours they trust. However, we chose not to include this item either individually or as part of a scale due to a high proportion of missing data (18%) for this item and poor internal consistency with other scale items. Despite this, and in order to explain the difference with other studies, we run further analysis with perceived trust both as a single item and as part of the ‘perceptions of neighbourhood quality’ scale. In both cases, perceived trust showed no association with CMD at neither individual nor ecological levels. So our conclusion is that the difference with other studies might be due to the large number of missing data and poor internal consistency with other scale items.

Interestingly, our study found that respondents in high incivilities neighbourhoods are at lower risk of experiencing CMD than respondents who live in low incivilities neighbourhoods. This contradicts literature which used similar independent and outcome variables [31] and more generally studies that used GHQ-28 as outcome variable [32]. The only plausible explanation, we could find is rooted in the process of statistical analysis. In moderation analysis, the effect of the physical environment is calculated when interaction terms are equal to zero. In this article, interaction terms were the measures of both cognitive and structural social capital as independent variables. Structural social capital, in particular, includes ‘social networks’ which is a measure of ‘social activity’ in the neighbourhood and has been found consistently negatively associated with CMD. Similarly, incivilities (e.g. broken glass, graffiti, vandalised facilities, broken windows, security measures) could be seen as a measure of ‘social activity’, albeit social activity that leads to damage rather than positive outcomes. Thus, a potential statistical explanation for this apparent ‘paradox’ might be found in the fact that the analysis considers ‘incivilities’ as a form of ‘social activity’ and as such associated to lower risk of CMD as analyses of ‘social networks’ have revealed. 

### Limitations

Although we introduced a longitudinal component to reinforce inferences on the causal relationship between types of social capital and CMD, this was limited to ecological rather than individual social capital as the sample populations for the two time points are different. Thus, this study includes almost all the major problems associated with cross-sectional studies. For instance, the problem of reverse causality might apply, particularly to the individual-level associations observed. Participants with less or no mental disorder are more likely to engage in social networks, and evidence from multilevel studies point to generally small differences in mental health across areas [19,33]. However, in order to examine this in the context of disadvantaged urban areas, we compared the GHQ-12 level of individuals in our sample with data from The British Household Panel Survey. We found that our sample has much better average mental health than the national average (Table S1). Thus, the reverse causality argument could well apply. An additional potential limitation might be a potential over-estimation in assessing the importance of social networks. The question included written communication between the respondent and friends/relatives. Written communication includes text, email, twitter to mention a few. Although these represent communication and help to create and/or maintain social networks, their frequency is very high, thus boosting the number of contacts substantially and affecting somewhat artificially a social network measure. 

### Policy implications

This study has emphasised that social networks might play an important role in limiting or protecting against CMD and for general health [34]. In considering some policy implications from this study, time banking deserves to be mentioned. Time banks are fundamentally based on building and maintaining social networks between people in the neighbourhood by mutual exchange of services using ‘time’ as a currency. By offering services, Time Bank members accumulate time credits which they can trade in purchasing other services from other members. Conversely, those receiving services accumulate debts which need to be repaid by offering services to other members. Some, albeit limited, evidence suggests that Time Banks improve individual’s mental health, particularly depression, by building networks of trust, reciprocity, and self-esteem [35,36]. 

### Future research

Beyond the policy applications of our finding, the importance of social networks for mental health suggests that future research should examine social networks in more depth. First, the measure of ‘social networks’ we used for our study only included extra-household contacts with relatives and friends rather than family which could conceivably offer an additional dimension to associations between social networks and mental health. Thus the interaction between family and extra-household networks could be examined as these are likely to be different and their associations with mental health also different. Second, our study showed that civic participation might be negatively associated to mental health. Future research should investigate civic participation in more detail, distinguishing between different forms of participation, not least because this is such an important part of what social capital is. Finally, we can only agree with previous studies [5] that highlighted the need to examine social capital longitudinally through a cohort study to minimise the effect of reverse causality.

## Supporting Information

Table S1 **GHQ-12 comparison between Well London sample and ‘Understanding Society’ (British Household Panel Survey 2009/10).**
(DOCX)
