# Supplementary material for: Types of Social Capital and Mental Disorder in Deprived Urban Areas: A Multilevel Study of 40 Disadvantaged London Neighbourhoods
Source: PLoS One. 2013 Dec 2;8(12):e80127. doi: 10.1371/journal.pone.0080127 (PMC3846561; doi:10.1371/journal.pone.0080127)
Supplement: Table S1 — GHQ-12 comparison between Well London sample and ‘Understanding Society’ (British Household Panel Survey 2009/10). (DOCX) [file pone.0080127.s001.docx]

Appendix 1

GHQ-12 comparison between Well London sample and ‘Understanding Society’ (British Household Panel Survey 2009/10)(*)

|  | GHQ-12 values | Well London sample (2011/12) | UK (2002/10) - mean |
| --- | --- | --- | --- |
|  | Scores | % | |
| No evidence of mental disorder | 0 or 1 | 86.4 | 67.0 |
| Some evidence of mental disorder | => 2 | 13.6 | 33.0 |
| Severe symptoms of mental disorder | 11 or 12 | 0.3 | 3.1 |

(*) For more information about ‘Understanding Society’

<http://www.understandingsociety.org.uk/design/features/bhps.aspx>
